# Correlation Between HLA-A, B and DRB1 Alleles and Severe Fever with Thrombocytopenia Syndrome

**DOI:** 10.1371/journal.pntd.0005076

**Published:** 2016-10-19

**Authors:** Shu-jun Ding, Yi Zhang, Xiao-mei Zhang, Xiao-lin Jiang, Bo Pang, Yong-hong Song, Jian-xing Wang, Yao-wen Pei, Chuan-fu Zhu, Xian-jun Wang, Xue-jie Yu

**Affiliations:** 1 Department of Viral Infectious Diseases Control and Prevention, Shandong Provincial Key Laboratory of Communicable Disease Control and Prevention, Shandong Center for Disease Control and Prevention, Jinan, Shandong Province, China; 2 HLA Lab,Blood Center of Shandong Province, Jinan, Shandong Province, China; 3 School of Public Health, Shandong University, Jinan, Shandong Province, China; 4 Department of Pathology, University of Texas Medical Branch, Galveston, Texas, United States of America; University of North Carolina at Chapel Hill, UNITED STATES

## Abstract

**Objective:**

Severe fever with thrombocytopenia syndrome (SFTS) is an emerging hemorrhagic fever caused by a tick-borne bunyavirus (SFTSV) in East Asian countries. The role of human leukocyte antigen (HLA) in resistance and susceptibility to SFTSV is not known. We investigated the correlation of HLA locus A, B and DRB1 alleles with the occurrence of SFTS.

**Methods:**

A total of 84 confirmed SFTS patients (patient group) and 501 unrelated non-SFTS patients (healthy individuals as control group) from Shandong Province were genotyped by PCR-sequence specific oligonucleotide probe (PCR-SSOP) for HLA-A, B and DRB1 loci.Allele frequency was calculated and compared using χ^2^ test or the Fisher's exact test. A corrected *P* value was calculated with a bonferronis correction. Odds Ratio (OR) and 95% confidence intervals (CI) were calculated by Woolf’s method.

**Results:**

A total of 11 HLA-A, 23 HLA-B and 12 HLA-DRB1 alleles were identified in the patient group, whereas 15 HLA-A, 30 HLA-B and 13 HLA-DRB1 alleles were detected in the control group. The frequencies of A*30 and B*13 in the SFTS patient group were lower than that in the control group (*P* = 0.0341 and 0.0085, *Pc* = 0.5115 and 0.252). The ORs of A*30 and B*13 in the SFTS patient group were 0.54 and 0.49, respectively. The frequency of two-locus haplotype A*30-B*13 was lower in the patient group than in the control group(5.59% versus 12.27%, *P* = 0.037,OR = 0.41, 95%CI = 0.18–0.96) without significance(*Pc*>0.05). A*30-B*13-DRB1*07 and A*02-B*15-DRB1*04 had strong associations with SFTS resistance and susceptibility respectively (*Pc* = 0.0412 and 0.0001,OR = 0.43 and 5.07).

**Conclusion:**

The host HLA class I polymorphism might play an important role with the occurrence of SFTS. Negative associations were observed with HLA-A*30, HLA-B*13 and Haplotype A*30-B*13, although the associations were not statistically significant. A*30-B*13-DRB1*07 had negative correlation with the occurrence of SFTS; in contrast, haplotype A*02-B*15-DRB1*04 was positively correlated with SFTS.

## Author Summary

Severe fever with thrombocytopenia syndrome (SFTS) is an emerging hemorrhagic fever caused by a tick-borne bunyavirus (SFTSV) in East Asian countries. The role of human leukocyte antigen (HLA) in resistance and susceptibility to SFTSV is not known. In this study, we investigated the correlation of HLA locus A, B and DRB1 alleles with the occurrence of SFTS. Our results have expanded the knowledge of the association of HLA genes with SFTS. Our study may be helpful to state the relationship between the occurrence of SFTS with HLA alleles or haplotypes and provide scientific basis for study on pathogenesis and vaccine development.

## Introduction

Sever fever with thrombocytopenia syndrome (SFTS) is an emerging infectious disease in China, South Korea and Japan [1–3]. SFTS is caused by a novel bunyavirus, SFTS virus (SFTSV), which is transmitted through tick bite [1,4–6]. Shandong Province is the second highest incidence area of SFTS in China. Since starting surveillance on SFTS patients in 2010, 761 SFTS cases were reported in Shandong Province from 2011 to 2014 with annual case fatality of 12.5% [7]. Number of reported SFTS cases was rising and SFTS inflict areas was expanding from the initial 6 cities in 2010 to 15 cities in 2014 among 17 cities in Shandong Province.

SFTS is a hemorrhagic fever disease with fever and thrombocytopenia as the main clinical manifestations. The body temperature of most SFTS cases usually exceeds 38°C. Over 70% of the patients have fever >39°C [8,9]. Patients often had headaches, muscle aches, gastrointestinal symptoms such as lack of appetite, nausea, vomiting, abdominal pain, diarrhea and hematochezia, leukopenia, liver and kidney dysfunction. The vast majority of patients has a good prognosis and recovered. On the contrary, some patients have a poor prognosis because of accompanied by basic diseases, older ages, the emergence of neuropsychiatric symptoms, bleeding tendency obviously and hyponatremia.

Those patients who had severe bleeding tendency and in critical condition might die of multiple organ failure [1]. The overall mortality rate of SFTSV infection is about 12%, ranging from 6.3% to 30.0% in previous studies [10,11]. A sustained serum viral load may indicate that disease conditions will worsen and lead to death [8]. In multivariate analysis, the odds for SFTS were 2.4~4.5 fold higher with patients who reported tick bites or presence of tick in the living area [12]. Our previous results revealed that age was the critical risk factor or determinant for SFTS morbidity and mortality [13]. However, the mechanism of susceptibility to SFTSV is not clear. There is no evidence on the role of T cells in the pathogenesis of SFTS because SFTSV is a newly discovered virus. Most studies have focused on humoral immunity and innate immunity of SFTSV. We are not aware any study on T cell immunity of SFTSV.

The human leukocyte antigens (HLA) were the human versions of the major histocompatibility complex (MHC) genes that were found in most vertebrates. The HLA genes encoded cell-surface antigen-presenting proteins, which regulated the immune system in humans and were essential elements for immune function. HLA was highly polymorphic and was significantly different in populations in different geography, ethnic and race [14–16]. HLA determined the individual differences in susceptibility to pathogens or diseases. Studies showed that HLA was related to ankylosing spondylitis, diabetes, psoriasis and other autoimmune diseases [17–19]. It also correlated to AIDS, hepatitis B and other viral infections [20–22], but the correlation between HLA polymorphism and SFTS had not yet investigated. In this study, we analyzed the frequency of three important HLA alleles and haplotypes comprised by these alleles in SFTS patients and healthy individuals to determine whether HLA alleles and/or haplotypes correlated to the occurrence of SFTS.

## Materials and Methods

### Ethics Statement

Subjects in the study were all adults. SFTS samples were collected for disease surveillance and disease diagnosis. Serum samples from healthy individuals (non-SFTS patients) who were volunteers of China Marrow Donor Program (CMDP) were collected for HLA matching. All samples from SFTS cases and healthy individuals were pre-existing relative to the start of the study, and were examined as anonymous samples. The study was approved by the Ethic Committee of Preventive Medicine of Shandong Center for Disease Control and Prevention (no. 2011–12). All infected adults subjects had signed a written informed consent document for collecting their serum specimen.

### Study sites

All patients and healthy persons were from Shandong Province, which located in the eastern coast of China between north latitude 34° 22.9 '- 38° 24.01' and longitude 114° 47.5 '- 122° 42.3'. The province consisted of 15.50% mountain area, 13.20% of hill area, 55% plains, 4.10% depression area, 4.40% lake plain, and 7.80% other area. Shandong's climate was temperate monsoon types with annual average temperature between 11°C and 14°C, annual average rainfall between 550 and 950 mm. As of December 2013, the resident population in the province was 97.33 million. Han Chinese was the dominant population with 0.70% ethnic minorities in the total population of Shandong Province.

### SFTS patients and healthy population

84 cases had whole blood specimens and were used in this study for HLA allele typing. The patient group consisted of 84 SFTS patients, who were diagnosed at local hospitals in 2013 in Shandong Province and reported to the China Information System for Diseases Control and Prevention. All SFTS patients were laboratory confirmed for bunyavirus, SFTS virus (SFTSV).The control group consisted of 501 unrelated healthy individuals (non-SFTS patients) who were volunteers of China Marrow Donor Program (CMDP) and their blood was available and obtained from the Blood Center of Shandong Province. All participants including SFTS patients and healthy individuals were native Han ethnic Chinese from Shandong Province.

### Human DNA extraction

Human blood DNA was extracted from whole blood using EZBead^TM^ whole blood DNA extraction kit (Texas BioGene Inc, Richardson, Texas). DNA concentration was 20–100ng/μL and DNA purity was 1.70 to 1.85 at OD260 / OD280.

### HLA genotyping

Patients and healthy individuals’ HLA-A, B and DRB1 alleles were genotyped using PCR-SSOP methods with low resolution LABType SSO Typing Tests (One Lambda Inc., Canoga Park, CA) according to the manufacturer’s instructions. The test results were analyzed with HLA Tools (One Lambda Inc., Canoga Park, CA).

### Data analysis

HLA allele frequencies (AF) were calculated and haplotype frequencies (HF) in patients and healthy individuals were estimated using the maximum-likelihood method with the expectation-maximization (EM) algorithm in the Arlequin V3.5 software. The frequency difference between the patient group and control group was compared using χ^2^ test or the Fisher's exact test. The extent of correlation of HLA alleles and haplotypes between the patient group and the control group was indicated by the odds ratio (OR), which was obtained by Woolf’s method. A corrected *P* value (*Pc*) was further to be calculated with a bonferronis correction by multiplying the *P*-value with the number of alleles tested for each locus.

## Results

### Patient information

In 2013, 296 SFTS cases were clinically reported in Shandong Province including 85 clinical diagnosed cases and 211 laboratory confirmed cases. 84 cases had whole blood specimens and were used in this study for HLA allele typing. The 84 confirmed cases were all Han ethnic Chinese from Shandong Province including 7.14% (6/84) death cases. Majority of patients were from Weihai City (65.48%, 55/84) and Tai’an City (17.86%, 15/84). The remaining 14 patients were from five other cities. Patients ranged from 28 years old to 84 years old with median age of 62 years old. Patients’ age distribution was summarized in Table 1. Majority of patients went to clinic for treatment within 5 days (58.33%, 49/84) to 10 days (86.90%, 73/84) after onset of illness.

**Table 1:** Age distribution in patient group.

Age group	Number of cases	Percentage %	Case fatality rate %
20~	1	1.19	0
30~	2	2.38	0
40~	10	11.90	0
50~	24	28.57	8.33(2/24)
60~	22	26.19	4.55(1/22)
70~	19	22.62	5.26(1/19)
≥80	6	7.14	33.33(2/6)
Total	84	100	7.14(6/84)

20~:including SFTS cases whose age was≥20 years old, but<30 years old.

The clinical manifestations of the patients included fever, dizziness, headache, nausea, vomiting, fatigue, muscle aches, cough, sputum and gastrointestinal symptoms of anorexia, diarrhea, and abdominal pain. The clinical manifestations of 52 SFTS cases with complete information were listed in Table 2.

**Table 2:** The main clinical symptoms and signs of 52 severe fever with thrombocytopenia syndrome cases.

Symptoms and signs	Number of cases	Frequency (%
Fever	51	98.08
Chills	21	40.38
Headache	15	28.85
Weakness	42	80.77
Body ache	24	46.15
Conjunctival hyperemia	2	3.85
Skin petechiae or ecchymosis	8	15.38
Bleeding gums	4	7.69
Loss of appetite	15	28.85
Nausea	27	51.92
Vomit	20	38.46
Haematemesis	0	0
stomach ache	8	15.38
Bloating	7	13.46
Diarrhea	16	30.77
Back pain	5	9.62
Lymphadenopathy	7	13.46

### HLA-A allele profiles in SFTS patients

A total of 11 HLA-A alleles were detected in the patient group and 15 HLA-A alleles were detected in the control group. Six HLA-A alleles (A*01, A*02, A*11, A*24, A*30 and A*33) were all found with a frequency greater than 5% in both the SFTS patient group and the control group, with a cumulative frequency of 86.30% and 84.43%, respectively. HLA-A locus was dominated by the A*02 allele with a frequency of 28.57% and 27.25% in the SFTS patient group and the control group, respectively. The next five most common alleles were A*24(17.*26%)*, A*11(16.*07%)*, A*33(10.*71%)*, A*30(8.*33%)* and A*01(5.*36%*) in the SFTS patient group, and A*30(14.*37%)*, A*11(13.*87%)*, A*24(12.*87%)*, A*33(10.*28%)* and A*01(5.*79%)* in the control group. Statistical analysis of the frequency of different HLA-A alleles in the patient and control groups indicated that the frequency of A*30 in the patient group (8.33%) was lower than that in the control group (14.37%) and the difference between two groups was noted (*P* = 0.0341, OR = 0.54, 95%CI, 0.30–0.96).However, A*30 did not reach statistically significant after Bonferroni correction(*Pc* = 0.5115). Other HLA-A alleles were not significantly different between the patient group and the control group (Table 3).

**Table 3:** Frequencies of HLA-A allele and odds ratio in SFTS patient and control groups (patients 2n = 168 control 2n = 1002).

Locus	Patients (2n = 168)		Control (2n = 1002)		*P*	OR	95% CI
	Count	AF(%)	Count	AF(%)			
A* 01	9	5.36	58	5.79	*0*.*8238*	0.92	0.45–1.9
A* 02	48	28.57	273	27.25	*0*.*7215*	1.07	0.74–1.53
A* 03	6	3.57	34	3.39	*0*.*9064*	1.05	0.44–2.55
A* 11	27	16.07	139	13.87	*0*.*4497*	1.19	0.76–1.86
A* 23	0	0	9	0.90	*0*.*4496*	NA	NA
A* 24	29	17.26	129	12.87	*0*.*1236*	1.41	0.91–2.19
A* 26	6	3.57	20	2.00	*0*.*9064*	1.05	0.44–2.55
A* 29	1	0.6	13	1.30	*0*.*6956*	0.46	0.06–3.51
A* 30	14	8.33	144	14.37	*0*.*0341*	0.54	0.30–0.96
A* 31	8	4.76	39	3.89	*0*.*5952*	1.23	0.57–2.69
A* 32	0	0	28	2.79	*0*.*0548*	NA	NA
A* 33	18	10.71	103	10.28	*0*.*8640*	1.05	0.62–1.78
A* 68	2	1.19	11	1.10	*0*.*7706*	1.09	0.24–4.94
A* 69	0	0	1	0.10	*0*.*3093*	NA	NA
A* 74	0	0	1	0.10	*0*.*3093*	NA	NA

OR, odds ratio; CI, confidence interval; NA, not applicable. A *P*-value less than 0.00333 is significant after correction for multiple comparison.

### HLA-B allele profiles in SFTS patients

For HLA-B, a total of 23 HLA-B alleles were identified in the SFTS patients and 30 HLA-B alleles were identified in the control group. The highest frequencies of HLA-B antigen specificities in the patient group were as follow: B*15(14.88%), B*40(13.1%), B*13(9.52%), B*51(9.52%), B*46(7.14%),B*35(5.95%) and B*44(5.36%),consisting of 65.47% of the HLA-B alleles of the patient group. In the control group, the most common alleles in descending order were B*13(17.66%), B*15(14.47%), B*40(11.38%), B*51(6.99%), B*44(6.39%) and B*35(5.29%) consisting of 62.18% of HLA-B alleles of the control group. In the patient groups, a 1.87 fold decrease was observed with HLA-B*13 allele compared to healthy controls (*P* = 0.0085, OR = 0.49, 95%CI = 0.29–0.84). Although we observed the negative association, B*13 did not reach statistically significant after Bonferroni correction (*Pc* = 0.252) (Table 4).

**Table 4:** Frequencies of HLA-B allele and odds ratio in SFTS patient and control group (patients 2n = 168, control 2n = 1002).

Locus	Patients (2n = 168)		Control (2n = 1002)		*P*	OR	95% CI
	Count	AF(%)	Count	AF(%)			
B*07	8	4.76	41	4.09	*0*.*6882*	1.71	0.54–2.55
B*08	2	1.19	8	0.80	*0*.*9537*	1.50	0.32–7.11
B*13	16	9.52	177	17.66	***0*.*0085***	0.49	0.29–0.84
B*14	0	0	2	0.20	*0*.*6676*	NA	NA
B*15	25	14.88	145	14.47	*0*.*8890*	1.03	0.64–1.64
B*18	0	0	3	0.30	*0*.*9091*	NA	NA
B*27	4	2.38	26	2.59	*0*.*9192*	0.91	0.32–2.66
B*35	10	5.95	53	5.29	*0*.*7246*	1.13	0.56–2.27
B*37	1	0.6	10	1.00	*0*.*9452*	0.59	0.08–4.67
B*38	1	0.6	30	2.99	*0*.*1255*	0.19	0.03–1.43
B*39	5	2.98	15	1.50	*0*.*2950*	2.02	0.72–5.63
B*40	22	13.1	114	11.38	*0*.*5203*	1.17	0.72–1.91
B*41	1	0.6	1	0.10	*0*.*6676*	5.99	0.37–96.30
B*42	0	0	1	0.10	*0*.*3072*	NA	NA
B*44	9	5.36	64	6.39	*0*.*6095*	0.83	0.40–1.70
B*45	0	0	2	0.20	*0*.*6676*	NA	NA
B*46	12	7.14	43	4.29	*0*.*1061*	1.72	0.89–3.33
B*47	0	0	3	0.3	*0*.*9091*	NA	NA
B*48	8	4.76	27	2.69	*0*.*1455*	1.81	0.81–4.04
B*49	0	0	1	0.1	*0*.*3072*	NA	NA
B*50	4	2.38	10	1.0	*0*.*2534*	2.42	0.75–7.81
B*51	16	9.52	70	6.99	*0*.*2434*	1.40	0.79–2.48
B*52	6	3.57	39	3.89	*0*.*8414*	0.91	0.38–2.19
B*54	5	2.98	35	3.49	*0*.*7330*	0.85	0.33–2.19
B*55	2	1.19	10	1.0	*0*.*8536*	1.20	0.26–5.50
B*56	1	0.6	1	0.1	*0*.*6676*	5.99	0.37–96.30
B*57	3	1.79	13	1.3	*0*.*8844*	1.38	0.39–4.91
B*58	6	3.57	43	4.29	*0*.*6664*	0.83	1.9700
B*67	0	0	12	1.2	*0*.*3115*	NA	NA
B*73	1	0.6	0	0	*0*.*0392*	NA	NA
B*81	0	0	3	0.3	*0*.*9091*	NA	NA

OR, odds ratio; CI, confidence interval; NA, not applicable. A *P*-value less than 0.00167 is significant after correction for multiple comparison.

### HLA- DRB1 allele frequencies

For HLA- DRB1 alleles, 12 alleles were detected in SFTS patients and 13 alleles were detected in the control group. The most prevalent DRB1 genes in the patient group were DRB1*15(20.83%), DRB1*07(14.29%), DRB1*04(11.9%), DRB1*12(10.12%), DRB1*13(6.55%), DRB1*14(5.95%) and DRB1*08(5.36%), which were found in 75% of SFTS patients. The most prevalent DRB1 genes in the control group were DRB1*15(19.76%), DRB1*07(17.37%), DRB1*12(9.98%), DRB1*04(8.58%), DRB1*13(7.78%), DRB1*08(6.39%) and DRB1*14(5.49%), which were found in 75.35% of healthy persons in the control group. DRB1*15 was the most common HLA- DRB1 allele in the patient group (20.83%) and in the control group (19.76%). There were no positive or negative associations of HLA-DRB1 alleles were observed between patient group and control group (Table 5).

**Table 5:** Frequencies of HLA-DRB1 alleles and odds ratio in SFTS patient and control group (patients 2n = 168, control 2n = 1002).

Locus	Patients (2n = 168)		Control (2n = 1002)		*P*	OR	95% CI
	Count	AF(%)	Count	AF(%)			
DRB1*01	3	1.79	27	2.69	*0*.*6701*	0.6566	0.20–2.19
DRB1*03	7	4.17	38	3.79	*0*.*8154*	1.1030	0.48–2.51
DRB1*04	20	11.90	86	8.58	*0*.*1651*	1.4393	0.86–2.41
DRB1*07	24	14.29	174	17.37	*0*.*3245*	0.7931	0.50–1.26
DRB1*08	9	5.36	64	6.39	*0*.*6095*	0.8296	0.40–1.70
DRB1*09	22	13.10	101	10.08	*0*.*2383*	1.3442	0.82–2.20
DRB1*10	0	0	11	1.10	*0*.*3511*	NA	NA
DRB1*11	8	4.76	46	4.59	*0*.*9221*	1.0391	0.48–2.24
DRB1*12	17	10.12	100	9.98	*0*.*9557*	1.0155	0.59–1.75
DRB1*13	11	6.55	78	7.78	*0*.*5758*	0.8300	0.43–1.60
DRB1*14	10	5.95	55	5.49	*0*.*8083*	1.0898	0.54–2.18
DRB1*15	35	20.83	198	19.76	*0*.*7473*	1.0686	0.71–1.60
DRB1*16	2	1.19	24	2.40	*0*.*4855*	0.4910	0.11–2.10
DRB1*01	3	1.79	27	2.69	*0*.*6701*	0.6566	0.20–2.19
DRB1*03	7	4.17	38	3.79	*0*.*8154*	1.1030	0.48–2.51

OR, odds ratio; CI, confidence interval; NA, not applicable. No alleles were significantly different between SFTS patients and healthy individuals.

### HLA haplotype profiles in SFTS patients

Table 6 shows the comparison of two-locus and three-locus haplotype frequencies in SFTS patient group and control group. For HLA-A-B, the HLA-A*30-B*13 haplotype was found with a lower frequency in SFTS patient group than in control group (5.59% versus 12.27%, *P* = 0.037,OR = 0.41, 95%CI = 0.18–0.96). It displayed association with SFTS resistance. But HLA-A*30-B*13 did not reach statistic significant after Bonferroni correction(*Pc* >0.05).For HLA-A-DR, no association was noted between two groups. Through statistical analysis of the frequency of HLA-B*-DR* haplotypes, haplotype B*15-DRB1*04 displayed association with SFTS susceptibility (*P* = 0.0224, OR = 2.95, 95%CI = 1.12–7.77). Otherwise, the difference was not statistically significant after multiple comparisons(*Pc*>0.05). Comparing the frequency of HLA-A*-B*-DR* haplotypes in the patient group and the control group, the results showed A*30-B*13-DRB1*07 and A*02-B*15-DRB1*04 had strong associations with SFTS resistance and susceptibility respectively (*Pc* = 0.0412 and 0.0001,OR = 0.43 and 5.07).Although there were differences of the other 5 three-locus haplotypes including A*02-B*46-DRB1*09, A*02-B*50-DRB1*07, A*02-B*40-DRB1*15,A*33-B*44-DRB1*07 and A*24-B*15-DRB1*04 among two groups, they did not reach statistic significant(*Pc*>0.05)(Table 6).

**Table 6:** Two-locus and three-locus haplotype frequencies and odds ratio in SFTS patient group and control group (patients 2n = 168, control 2n = 1002).

Haplotype	HF (Patient,%)	HF (Control,%)	*P*	OR	95%CI
A*02-B*15	6.69	5.32	0.5229	1.32	0.56–3.10
A*30-B*13	5.59	12.27	0.0347	0.41	0.18–0.96
A*24-B*40	4.77	2.21	0.1645	2.22	0.70–7.02
A*11-B*15	4.56	3.25	0.4768	1.46	0.51–4.20
A*02-B*46	4.42	2.75	0.3574	1.65	0.56–4.86
A*11-B*35	4.21	0.93	0.0578	3.71	0.87–15.77
A*33-B*44	4.02	3.55	0.8842	1.08	0.36–3.24
A*24-B*51	3.85	1.94	0.3568	2.21	0.63–7.77
A*02-B*40	3.45	4.08	0.6744	0.78	0.24–2.50
A*02-B*48	3.45	1.20	0.2914	2.94	0.65–13.35
B*15-DRB1*04	6.78	2.40	0.0224	2.95	1.12–7.77
B*13-DRB1*07	5.67	10.97	0.0653	0.49	0.23–1.06
B*40-DRB1*15	4.26	3.16	0.5008	1.41	0.52–3.87
B*46-DRB1*09	3.45	1.21	0.2241	3.05	0.72–12.94
B*44-DRB1*07	3.35	2.30	0.9025	1.29	0.40–4.13
B*58-DRB1*03	2.87	1.63	0.5406	1.82	0.52–6.38
B*35-DRB1*15	2.72	1.42	0.6372	1.81	0.45–7.35
B*15-DRB1*15	2.41	3.19	0.9263	0.79	0.24–2.61
B*48-DRB1*15	2.39	1.08	0.4305	2.42	0.54–10.97
B*40-DRB1*09	2.30	2.55	0.7788	1.02	0.30–3.55
A*02-DRB1*09	9.54	3.70	0.0743	2.47	0.89–6.85
A*02-DRB1*04	7.08	2.46	0.0956	3.34	0.95–11.73
A*11-DRB1*15	6.32	2.45	0.19	2.84	0.78–10.30
A*11-DRB1*12	5.09	3.33	0.7036	1.54	0.46–5.18
A*02-DRB1*15	5.03	7.20	0.477	0.68	0.24–1.97
A*24-DRB1*14	4.02	0.91	0.238	3.74	0.67–20.81
A*30-DRB1*07	3.73	9.74	0.1004	0.4	0.13–1.23
A*02-DRB1*12	3.45	3.97	0.9715	0.77	0.19–3.05
A*33-DRB1*13	3.15	2.79	0.7973	1.09	0.26–4.67
A*03-DRB1*07	2.87	0.54	0.2562	5.59	0.57–54.44
A*30-B*13-DRB1*07	4.60	9.93	0.0002	0.43	0.27–0.68
A*02-B*15-DRB1*04	4.02	0.81	0.0000	5.07	2.50–10.28
A*02-B*46-DRB1*09	3.45	1.24	0.0009	2.92	1.51–5.67
A*33-B*44-DRB1*07	2.87	1.39	0.0329	2.06	1.05–4.06
A*02-B*40-DRB1*15	2.30	2.11	0.0111	2.74	1.22–6.15
A*02-B*50-DRB1*07	2.30	0.49	0.0005	4.46	1.79–11.16
A*24-B*15-DRB1*04	2.30	0.53	0.0005	4.46	1.79–11.16
A*33-B*58-DRB1*03	2.30	1.37	0.16	1.69	0.81–3.52
A*11-B*15-DRB1*12	2.16	1.59	0.5105	1.28	0.61–2.69
A*02-B*15-DRB1*15	1.72	1.71	0.9625	0.98	0.44–2.18

F, frequency; OR, odds ratio; CI, confidence interval. Alleles underlined were significantly different between SFTS patients and healthy individuals.

*P*-values less than 0.00045,0.00037,0.0005 and 0.00024 are significant for two-locus haplotypes HLA-A-B,HLA-B-DR,HLA-A-DR and three-locus haplotypes HLA-A-B-DR respectively after correction for multiple comparison.

## Discussion

HLA are cell surface transmembrane glycoproteins and these glycoproteins can bind peptides from inside and outside the cells to form HLA-polypeptides; antigen presenting cells transfer the polypeptide complex to T cells, which stimulates T cell’s differentiation and development, triggering immune response and adjusting the intensity of the immune response. Therefore, HLA determine the outcome of the infection of pathogenic microorganisms. HLA genes are divided into three categories: HLA class I genes encoded HLA molecules are widely distributed in the surface of nucleated cells; HLA class II genes encoded molecules are mainly distributed in the antigen presenting cells and activated T cell surface; HLA class III genes encode complement components. We analyzed the association of SFTS with human leukocyte antigens (HLA) because HLA correlates to AIDS, hepatitis B and other viral infections, but the correlation between HLA polymorphism and SFTS has not yet investigated.

SFTS is an emerging infectious disease and has been reported in 23 provinces in China with most cases came from central China including Henan, Shandong, Hubei, Anhui, Liaoning, Zhejiang and Jiangsu provinces. Most cases (88.3%) are famers and the distribution of the disease is associated with geography [7]. Clinical symptoms of SFTS include subclinical infection, mild, severe and fatal infections. The mechanisms of pathogenesis of different clinical symptoms are not clear. In this study we analyzed the correlation of HLA alleles with the occurrence of SFTSV infection. We selected 84 cases of SFTS patients and 501 healthy individuals as control to genotype the HLA-A, B, DRB1 alleles.

Song et al. reported that A*02 was the most common allele with a frequency of 28.86% in northern Han Chinese population [23]. Our results also confirmed it. The HLA-A*02 has the highest frequency of 28.57% in patient group and of 27.25% in control group. But there was no difference between two groups. The results showed the frequencies of HLA-A*30 and HLA-B*13 were lower in SFTS patient group than in control group, indicating that HLA-A*30 and HLA-B*13 may confer resistance to SFTS, although no significant differences were observed after Bonferroni correction. Alleles A*23, A*32, A*69, A*74, B*14, B*18, B*42, B*45, B*47, B*49, B*67, B*81 and DRB1*10 were not detected in the patient group. A*32 has a high frequency in the control group, but was not detected in the patient group. Similarly, B*67 and DRB1*10 also had high frequency in the control group, but were not detected in patient group. The frequency of these alleles were not significantly different between the two groups and their difference between the two groups was most likely caused by small sample size of the patient group, which need to be further explored for their correlation with SFTS by increasing SFTS patient sample size in future study.

In northern Han Chinese population, the most common HLA-A-B haplotypes (HF>0. 0300) were A* 30-B* 13, A* 02-B* 46, A* 33-B* 58, A* 33-B* 44, A* 02-B* 40[20]. We also found these HLA haplotypes were most common in the SFTS patient group and in the control group except for HLA-A* 33-B* 58. Through statistical analysis of the frequency of HLA-A-B haplotypes, we found that A*30-B*13 was less common in the patient group than in the control group (*P* = 0.0347, OR = 0.41, 95CI, 0.18–0.96) and the frequencies of these displayed HLA-A-B and HLA-A-DR haplotypes were not significantly different between the patient group and the control group by statistic analysis.

Moreover, haplotype A*30-B*13-DRB1*07 was significantly less commonly distributed in the SFTS patients group (*P* = 0.0002,*Pc* = 0.0412,OR = 0.43), indicating the haplotype negatively related to the incidence of SFTS as well as Zhang et al. reported that HLA haplotype A*30-B*13-C*06 confers HIV-1 infected patients with a long-term non-progressing condition [24]. Miao et al. showed that the frequency of HLA-B*13:*01*:*01G* increased significantly in HBsAg clearance group than that in the persistent group (8.57%versus 3.46%, *P* = 0.0004, *OR* = 2.62, 95% CI: 1.51–4.54) [25]. Chiewsilp et al. also reported a negative relationship for HLA-B*13 with dengue shock syndrome (DSS) and/ or dengue hemorrhagic fever (DHF)[26].These studies suggested that B*13 allele and haplotype A*30-B*13-C*06 is a protective factor against AIDS and hepatitis B, respectively. Our results and previous results indicate that HLA-A*30, HLA-B*13*, and A*30 -B*13* haplotypes play important roles in the outcome of viral infection. However, the role of HLA-B*13 in dengue virus infection is not the same. Appanna et al. demonstrated that HLA-B*13 is probably associated in dengue hemorrhagic fever susceptibility [27]. The specific mechanisms of HLA-B*13 in different viral infections need to be further studied.

On the other hand, another three-locus haplotypes A*02-B*15-DRB1*04 showed strong associations with SFTS susceptibility(*Pc* = 0.0001).It suggested that this haplotype might have caused individuals more susceptible to SFTS. Using low resolution typing method, we preliminary showed that A*30 and B*13 might have negative correlation with the occurrence of SFTS. Our results have expanded the knowledge of the association of HLA genes with SFTS. Our study may be further expanded by increased sample size and using high resolution typing method to verify the correlation between HLA -A*30 and B*13 alleles and SFTS and identify susceptible genes. Currently, there is no vaccine for SFTSV. Our work tried to disclose the association between SFTS and HLA. Identifying SFTS associated HLA alleles will potentially allow to define the SFTSV epitopes that are restricted by the specific HLA alleles. These HLA restricted epitopes of SFTSV (especially CTL epitopes) may be incorporated into vaccine design to prevent SFTSV infection[28,29].

## Supporting Information

S1 Checklist STROBE checklist. (DOCX)
